# Eating Habits and Body-weights of Students of the University of Belgrade, Serbia: A Cross-sectional Study

**DOI:** 10.3329/jhpn.v31i3.16824

**Published:** 2013-09

**Authors:** Tatjana Gazibara, Darija B. Kisic Tepavcevic, Aleksandra Popovic, Tatjana Pekmezovic

**Affiliations:** ^1^Institute of Epidemiology, Faculty of Medicine, University of Belgrade, Visegradska 26A, Belgrade 11000, Serbia; ^2^Faculty of Sport and Physical Education, University of Belgrade, Blagoja Parovica 156, Belgrade 11000, Serbia

**Keywords:** Nutritional status, Prevalence, Students, Serbia

## Abstract

The purpose of this survey was to quantify the prevalence of overweight and obesity among a sample of students in Belgrade University, Serbia and to describe their main eating habits. A total of 1,624 questionnaire responses were analyzed (response rate 97.3%). The students were recruited during mandatory annual check-ups in April-June 2009. All subjects completed the questionnaire; height (in cm) and weight (in km) were measured by two physicians. Results were assessed statistically. Almost every fourth male student was overweight. Strikingly, 15% of female students were underweight. Highly-significant difference was found between average body mass index (BMI) of male and female students (F=317.8, p=0.001). Students’ BMI did not correlate with average family income or with the frequency of taking breakfast (p=-0.064, p=0.152 for males and ρ=0.034, p=0.282 for females respectively). There is a growing demand for global health strategies which would encourage healthy body-image and figure; thus, these initiatives should mobilize the society on a national and international level.

## INTRODUCTION

Meal habits usually depend on lecture schedules attended by students and availability of food inside or in the vicinity of the university campus. As a result of the expansion in the fast-food market and lack of appropriate food courts, students usually face meal skipping, inadequate variety of foods, and snacking (1). Despite a plethora of publications on habits of university students and their health in the available literature, there has been no evaluation of nutritional status of this population in the Republic of Serbia. The purpose of this study was to quantify the prevalence of overweight and obesity among a sample of students in Belgrade University and to describe their main eating habits. This study is part of a larger analytical investigation among Belgrade University students, which estimated factors associated with quality of life (2).

## MATERIALS AND METHODS

### Subjects

Participants were recruited from the University of Belgrade, in the Republic of Serbia. The University of Belgrade, with 89,482 students, is the biggest and the oldest institution for higher education in the Balkan region. The university consists of 31 faculties divided into four sections: social sciences and humanities, medical sciences, nature sciences and mathematics, and technology and engineering sciences. The study was conducted in the Student Public Health Center in Belgrade, Serbia, in April-June 2009. A total of 1,669 consecutive students were recruited during mandatory annual check-ups. Forty-five students declined to fill the questionnaire, rendering a response rate of 97.3%. Thus, 1,624 questionnaire responses were analyzed. The inclusion criteria were paper-proof of enrollment in the Belgrade University. The number of recruited students represented about 1.8% of all Belgrade University students. Ethical approval for the study was obtained from the Ethics Committee of the Faculty of Medicine, University of Belgrade. Participation in this study was voluntary and anonymous. An informed consent form was signed by each student who agreed to participate.

### Instruments

All subjects completed the questionnaire which comprised demographic data, social status, place of residence during university schooling, regularity in breakfast-taking, frequency of consumption (meat and coffee), and portion-sizes (fruits and vegetables) consumed daily. One portion of fruit was defined as one medium-sized fruit (such as apple, orange, banana, and pear). One portion of vegetables was defined as ½ cup of raw, cooked, canned, or frozen vegetables. Height (in cm) and weight (in kg) were measured in each participant by two physicians (TG and AP, the first and third author of this artcile) before distributing the questionnaire. Body mass index (BMI) was calculated as the ratio of body mass in kg and the square of height in metre. Division of BMI into four categories was done according to the Centers for Disease Control and Prevention (CDC) guidelines: underweight <18.5, normal weight 18.5 to 24.9, overweight 25 to 29.9, and obese >30.

### Data analysis

Differences in analyzed parameters were assessed by using chi-square test and ANOVA. Bonferroni post-hoc test for locating differences between multiple groups was performed. Spearman's correlation coefficient (ρ) was used for investigating the association between the variables.

## RESULTS

Basic demographic data were shown elsewhere (2). Male students were, in average, significantly taller and weighed more than females (BMI 23.4±2.7 and 21.0±2.6 respectively, p<0.001). Mean BMI across genders varied within the normal weight range. According to BMI categories, three-quarters had normal weight (Table 1). Almost every fourth male student was overweight. In contrast, 15% of females were underweight. Obese students were the least common category with prevalence of only 1.5%. Students’ BMI did not correlate with average family income or with the frequency of taking breakfast (ρ=-0.064, p=0.152 for males and ρ=0.034, p=0.282 for females respectively). Mean BMI did not differ between those who ate breakfast and those who skipped it (21.8 vs 21.9, F=0.092, p=0.912). In addition, BMI did not differ according to students’ residence (with parents/alone/student dormitory, F=1.065, p=0.363). More than half of the students (57.3%) ate their breakfast regularly. One-third of subjects drank coffee every day, opposed to 27.3% who never drank it. Majority of students (80.1%) ate meat at least two times a week. Half of them ate one portion of fruits and vegetables daily (Table 2). There were no gender differences between consumption of fruits, vegetables, and meat (χ^2^=11.5, p=0.650).

**Table 1.** Distribution of students according to BMI categories

Weight status	Males (%)	Females (%)	Total (%)
Underweight*	19 (2.5)	134 (15.3)	153 (10.6)
Normal†	555 (73.8)	679 (77.9)	1,234 (76.4)
Overweight‡	165 (22.0)	46 (5.3)	211 (11.5)
Obese$	13 (1.7)	13 (1.5)	26 (1.5)
p value	0.001	0.001	

*BMI <18.5;

^†^BMI 18.5-24.9;

^‡^BMI 25-29.9;

$BMI ≥30

**Table 2.** Distribution of students according to daily consumption of fruits and vegetables

Daily portions	Fruits		Vegetables	
	Number	Percentage	Number	Percentage
Part of one	5	0.3	3	0.2
One	756	46.5	754	46.4
Two	367	22.7	418	25.6
Three	144	8.9	159	9.8
Four	33	2.0	19	1.2
Five	29	1.8	14	0.9
Six	2	0.1	1	0.1
Missing	288	17.7	256	15.8

## DISCUSSION

Most of the Belgrade University students were of normal nutritional status. Similar mean BMI was documented in students of different cultural backgrounds (3,4). Obese students accounted for only 1.5% in our sample. During 1997-2007, eating habits in the population of Serbia have been associated with irregular meals, decrease in fruits and vegetables consumption, along with related augmented intake of high-energy ingredients. In 2006, only half of the adults had had three regular meals per day while more than half were overweight (5).

In contrast, striking overall prevalence of underweight among Serbian students was recorded to be 10.6%. Proportion of females who were underweight was even higher (15.3%). Bodily proportions and BMI are certainly subject to emotional and physical well-being (6), which is particularly addressed in concomitant eating disorders (7). Also, university setting may have an important role in weight loss in the young adults as well. Academic pressures and time limits might often prevent students from regular meal consumption, leading to unintentional weight loss. Another possible explanation for such a high prevalence of underweight in female students might be related to eating disorders.

In this study, BMI in students did not differ among diverse place of residence during university schooling, implicating that parental involvement in meal regularity does not have impact upon weight change. On the other hand, individuals who take care of meal frequency on their own, accomplish this in a responsible manner without experiencing dramatic weight losses or gains. Survey by Milosevic *et al*. (8) revealed that the motives of primary food choice in general population in Serbia are sensory appeal and price. Staple foods in this region include potatoes and meat, usually that of pork. This reflects the prevalence of daily meat consumption by students (40%). Eighty percent of the subjects ate meat at least 2 times a week. Almost half ate two portions of fruits and vegetables daily, which, along with meat, expands the variety of daily food to a satisfactory level. In the Belgrade University, each faculty has its own cafe where students can spend time during the lecture breaks, however, without possibility of taking a proper meal. In our study, more than half of the students ate their breakfast regularly. The importance of taking breakfast was emphasized because of the local circumstance that university schooling schedule covers the period from 8 am to 6 pm. Therefore, it is crucial that students have regular morning meal before the start of daily duties. Additionally, in the Belgrade University, there were no organized lunch breaks between classes. Being the largest urban area of the country, Belgrade has recently been experiencing expansion of the food market that enables students to eat outside the faculty area. Such organization with higher-education facilities (i.e. faculty buildings in the centre of the city) differs, to a great extent, from the isolated campus structure in the USA. Consequently, recent investigation in California, USA (9), has documented a decrease in fruit and vegetable intake after starting college and living on-campus.

Since the students were filling in the questionnaire independently, we may consider reliability of data and potential information as well as recall bias. Additionally, there were no food intake diaries to quantify exact consumption of the ingredients.

### Conclusions

Nutritional interventions among students have already shown positive results (10). Therefore, promotion of healthy food consumption, with abundant fibres, whole grains, dairy products, and low energy-dense foods is needed. In addition, there is a growing demand for global health strategies which would encourage healthy body-image and figure; thus, these initiatives should mobilize the society on a national and international level.

## ACKNOWLEDGEMENTS

This investigation was supported by the Ministry of Science of the Republic of Serbia (Grant No. 175087).
